# Influence of Leukocyte- and Platelet-Rich Fibrin (L-PRF) in the Healing of Simple Postextraction Sockets: A Split-Mouth Study

**DOI:** 10.1155/2015/369273

**Published:** 2015-07-26

**Authors:** Gaetano Marenzi, Francesco Riccitiello, Mariano Tia, Alessandro di Lauro, Gilberto Sammartino

**Affiliations:** ^1^Division of Oral Surgery and Implantology, Department of Neurosciences, Reproductive and Odontostomatological Sciences, University of Naples “Federico II”, Via Pansini 5, 80131 Naples, Italy; ^2^Department of Neurosciences, Reproductive and Odontostomatological Sciences, University of Naples “Federico II”, Via Pansini 5, 80131 Naples, Italy; ^3^Private Practice, Via Austria 6, Sant'Antimo, Naples, Italy

## Abstract

The aim of this study was to evaluate the effects of leukocyte- and platelet-rich fibrin (L-PRF) on the pain and soft tissue healing after tooth extractions. Twenty-six patients (9 males and 17 females) were treated with multiple extractions (2 to 8), with a total of 108 extractions. This was an exploratory single blinded randomized clinical trial with a split-mouth design. The pain after the surgery was assessed in each patient by the VAS scale (1 to 10) at intervals of 24-48-72-96 hours. The soft tissue healing was clinically evaluated at 3, 7, 14, and 21 days after surgery by the same examiner surgeon, using the modified Healing Index (4 to 12). The mean value of postextraction pain was 3.2 ± 0.3 in the experimental sides and 4.1 ± 0.1 in the control sides. After 7 days from the extractions, the values of modified Healing Index in the experimental and control groups were, respectively, 4.8 ± 0.6 and 5.1 ± 0.9. The use of L-PRF in postextraction sockets filling can be proposed as a useful procedure in order to manage the postoperative pain and to promote the soft tissue healing process, reducing the early adverse effects of the inflammation.

## 1. Introduction

Many studies revealed that platelet concentrates for surgical use can be used as efficient adjuvants for tissue repair [1–5]. The growth factors (particularly platelet-derived growth factors (PDGF), transforming growth factors (TGF-*β*), and vascular endothelial growth factors (VEGF)) and the other molecules (fibrinogen, fibronectin, and vitronectin) contained in platelets (*α*-granules) give to these products the ability to modulate many phases of the healing process like the hemostasis and the neoangiogenesis [6]. The clinical results of these products are interesting but remain quite mixed and controversial in the literature, depending on the kind of preparation [7–10]. Platelet concentrates are classified into 4 main families depending on their leukocyte and fibrin content: pure platelet-rich plasma (P-PRP), leukocyte- and platelet-rich plasma (L-PRP), pure platelet-rich fibrin (P-PRF), and leukocyte- and platelet-rich fibrin (L-PRF) [11]. Each family of products has different aspect, biological content, and potential application [12].

The PRPs were already tested in many oral surgery applications, with mixed results depending on the kind of preparations [13–16]. Numerous protocols have attempted to optimize the preparation of the autologous factors, using various performances standards and centrifugation parameters [17, 18]. Several authors demonstrated the effectiveness of some PRP types during tooth extractions to stimulate soft tissue healing and wound control [19, 20] and in prevention of postoperative bleeding in anticoagulated patients undergoing oral surgery. However, these PRP techniques remain quite complex and expensive on a daily use basis, and their use may not be justified for daily oral surgery applications [13, 14].

On the other hand, L-PRF represents a more recent generation of platelet concentrates. The development of L-PRF is very significant in oral and maxillofacial surgery, with many validated applications in periodontal surgery [13, 21] and implant dentistry [14–22]. L-PRF is easy and inexpensive to prepare for frequent use in private practice, and it exists in the form of L-PRF clots or membranes (after compression). The membrane releases a significant quantity of autologous growth factors (particularly PDGF-AB, TGF*β*, and VEGF) [23], cytokines, and healing proteins (fibronectin, etc.) during more than 7 days in vitro [24], while other platelet gels dissolve in vitro in 3 days [12]. In another study, when compared with a procedure for platelet-rich plasma (PRP), L-PRF released more than 15-fold VEGF and more than 2-fold TGF*β*1 [25]. According to the literature, L-PRF was a useful tool in postextraction hemostasis control [26] and in prevention of hemorrhagic complications in cardiopathic patients [27].

The aim of this study is to evaluate the effectiveness of L-PRF to improve the soft tissues healing and to reduce pain after tooth extractions.

## 2. Materials and Methods

### 2.1. Study Population

From January 2012 to July 2013 at the Unit of Oral Surgery and Implantology of the University of Naples “Federico II,” 26 healthy patients were selected, including 9 males and 17 females with a mean age of 53 ± 4 years. The selected patients were nonsmokers or light smokers (<5/day); they did not have systemic diseases that could interfere with the healing process (such as diabetes, liver disease, heart disease, or immune-disorders) or diseases of the oral mucosa. The study was designed as a prospective split-mouth trial on patients who needed bilateral paired dental extractions; on the side chosen to be the study side, the sockets were filled with L-PRF, whereas on the other side (control), they were allowed to undergo natural healing. Test and control sites were chosen with coin toss randomization. This cross-sectional split-mouth research study was conducted in accordance with the requirements of Helsinki Declaration of 1975 as revised in 2008. Patients were verbally informed about the sample to be taken and gave their written consent. The patients who did not sign the agreement and also the patients with poor oral hygiene, patients with local infections of the soft tissues, patients undergoing bisphosphonates therapy, patients irradiated to the jaws in the past, and patients with psychiatric illness or pregnant patients were excluded from the study.

### 2.2. Surgical Procedure

**Figure 1:**
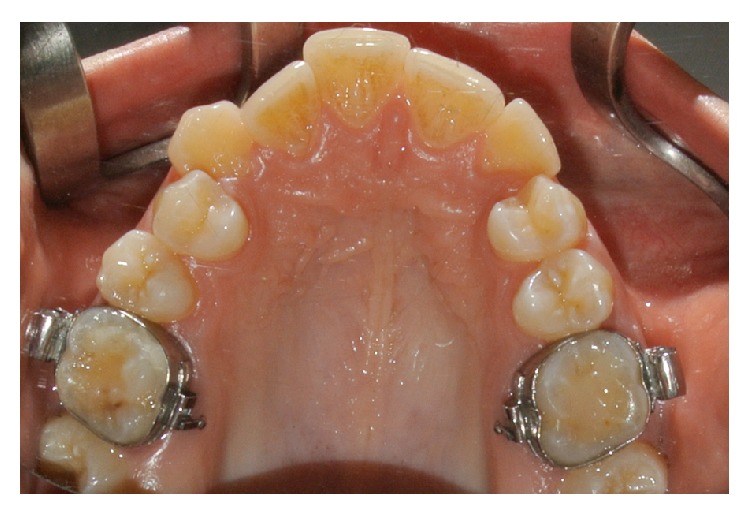
Presurgery clinical occlusal view. For orthodontic motive, this patient needed the bilateral extraction of the upper first premolars.

**Figure 2:**
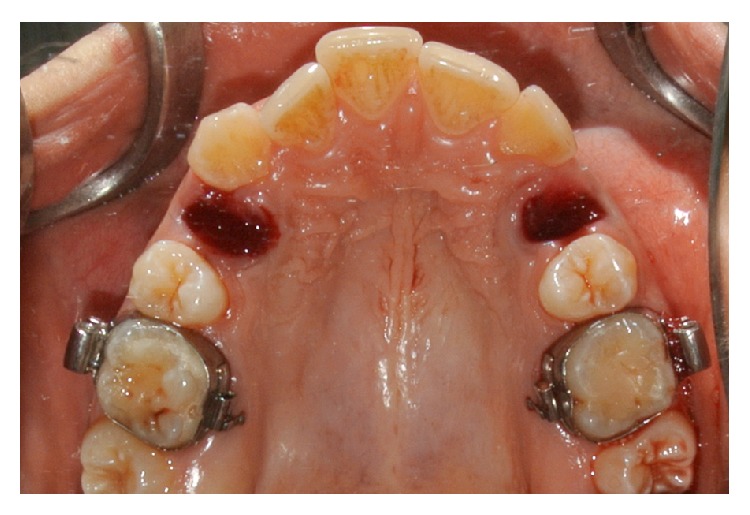
The postextraction sockets.

**Figure 3:**
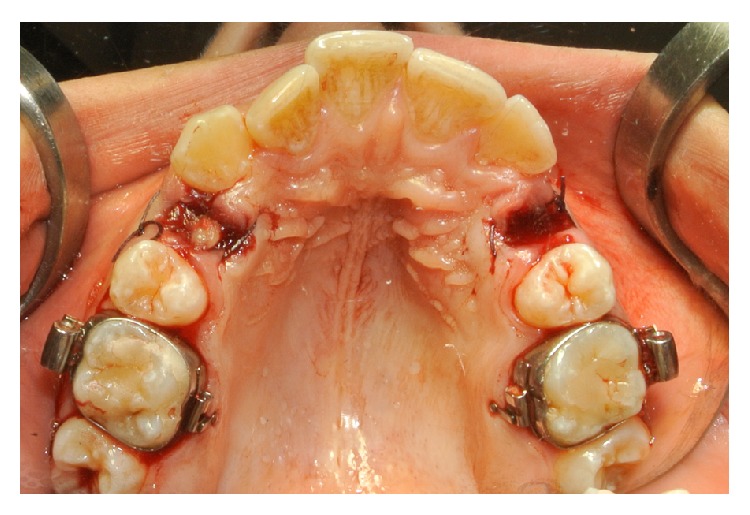
The upper right socket (study site) was filled with L-PRF, while the upper left socket (control site) had to follow natural healing. Both sites were sutured.

An alveolar nerve block infiltration was administrated with local or regional anesthesia, depending on the dental arch, using 2% mepivacaine. Mepivacaine does not contain epinephrine, so it was used to prevent restriction of the blood supply. To prevent interference with the healing process, no intraligamentous or intrapapillary infiltration was made. The teeth were extracted in a nontraumatic manner without elevation of full-thickness flaps and preserving the buccal and lingual walls of the alveolar sockets in order to minimize the possible trauma and to give adequate support to the L-PRF filling (Figures 1 and 2). All extraction sites were simple with alveolar walls preserved. All alveolar sockets were sutured with a 3/0 Vicryl (Ethicon/Johnson & Johnson, Somerville, NJ, USA). Each patient also served as the control (split-mouth design): a socket was treated with L-PRF application (study socket) whereas the other (control socket) had to undergo natural healing by clot formation without socket filling (Figure 3). The number of paired extractions per patient ranged from 2 to 4 for a total of 108 extractions. Indication for tooth extraction included root or crow fractures, residual roots, no restorable caries, periapical granuloma, and orthodontic reasons. Patients showing anatomic and pathologic conditions not comparable between the study and control sites were excluded as study subject. Antibiotic prophylaxis was undertaken (amoxicillin 875 mg and clavulanic acid 125 mg) starting 2 days before surgery up to 3 days after it.

### 2.3. L-PRF Preparation Protocol

The L-PRF was prepared through a single centrifugation of blood according to the protocol of Dohan Ehrenfest et al. (now marketed as Intra-Spin L-PRF kit, Intra-Lock, Boca-Raton, FL, USA) for a period of 12 minutes at 2700 rpm. Blood was taken in 9 mL tubes, 30 minutes before the surgery, immediately centrifuged, and used for the filing of the experimental sites. The total amount of blood collected (from 18 mL to 54 mL) was related to the number of tooth extractions, in order to obtain the complete filling of the sockets with the L-PRF. After centrifugation, each L-PRF clot was separated from the portion of red blood cells (red thrombus), obtaining a fibrin clot with a red small portion in order to include the “buffy” coat richer in large leucocytes [24]. The L-PRF clot was condensed and modeled on a sterile surgical plate before the application in the sockets [24].

L-PRF was used within 60 minutes after the preparation. It was accurately positioned in the extraction sites and stabilized with a resorbable suture. In the control sites, the same suture was used (Figure 3). All patients were advised to follow soft and liquid diet, avoiding hot food in the following hours. In all cases, the sutures were removed after one week.  Table 1 reports the number and the type of paired extractions per patient.

### 2.4. Study Variables and Statistical Methods

The predictor variable was the treatment group status: L-PRF versus control socket. The outcome variables of interest were as follows: pain, postsurgical complications to soft and hard tissues, and the Healing Index modified.

A 10-point visual analog scale (VAS) with a score of 0 that equals “no pain” and a score of 10 that equals “very severe pain” was used by the same patient to assess the postoperative pain at 24, 48, and 72 hours. Between groups comparisons for VAS outcomes were carried out by means of univariate analysis of variance, considering the group (i.e., PRF versus CTR) and the recording time point as factors and VAS score as dependent variable.

The quality of the socket soft tissue healing was clinically evaluated at 3, 7, 14, and 21 days after surgery by an examiner surgeon, using the Healing Index modified [28] which involved 3 scoring levels for each of the four parameters considered: bleeding, suppuration, tissue color, and consistency of the healing tissue. The scoring scale ranged from 4, corresponding to excellent healing, to 12, indicating severely impaired healing. The Wilcoxon signed-rank test for comparison of 2 correlated samples matched pairs with the level of significance predetermined at 0.05 was used.

## 3. Results

All patients completed the study. No cases of bleeding, infection, alveolar osteitis, or other surgical complications were reported.

Regarding the postextraction pain, patients enrolled in the study reported a mean value of the study sites of 3.2 ± 0.3, which is lower (*P* < 0.0001) than the mean value of the control sites (4.5 ± 0.7), with a statistical difference average of 0.9 ± 0.3. The VAS score was nearly equal for the 2 sides after 4 days (decreasing to 0).

**Figure 4:**
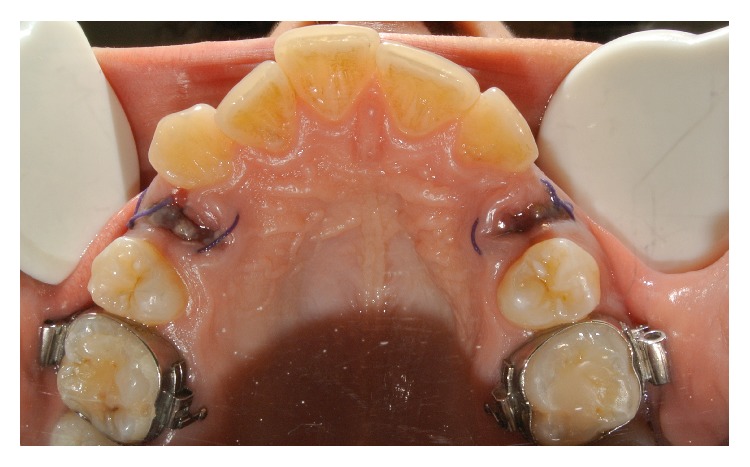
Clinical occlusal view 3 days after surgery. In the study site, the epithelialization process was more advanced than in the control site. In the study site, the inflammatory reaction was reduced.

**Figure 5:**
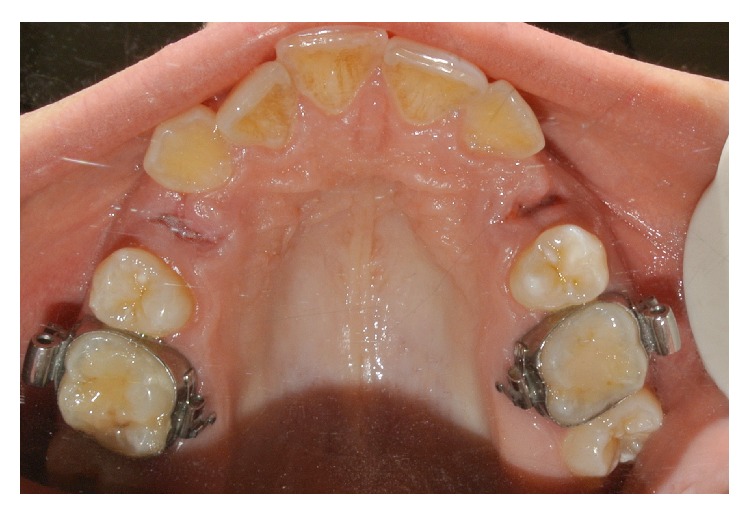
Clinical occlusal views 7 days after surgery. The sutures were removed. Both postextraction socket cavities presented a decreased volume and appeared epithelialized.

Results concerning the healing of the socket are reported in Table 2. Comparisons between values relative to the study and control sides showed better healing and faster socket closure for the side treated with L-PRF, with differences statistically significant at days 3 and 7 (Figures 4 and 5).

## 4. Discussion

**Figure 6:**
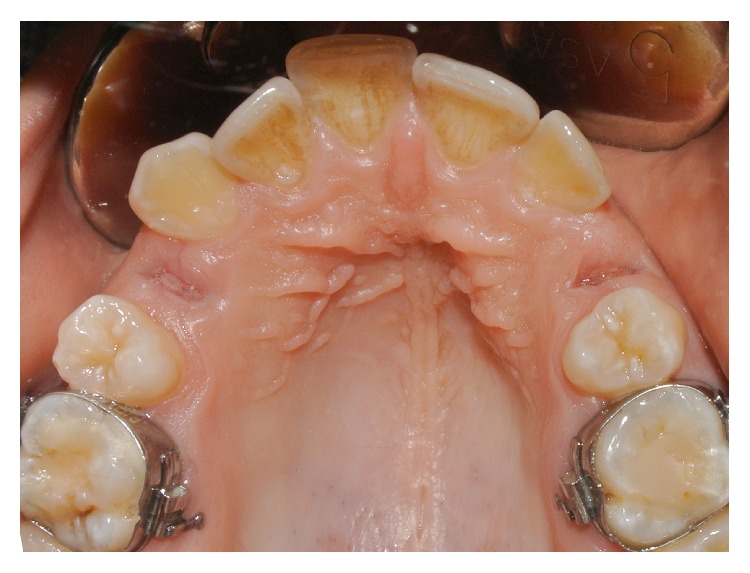
Clinical follow-up at 14 days after surgery. Both postextraction sockets were completely closed with the soft tissues.

**Figure 7:**
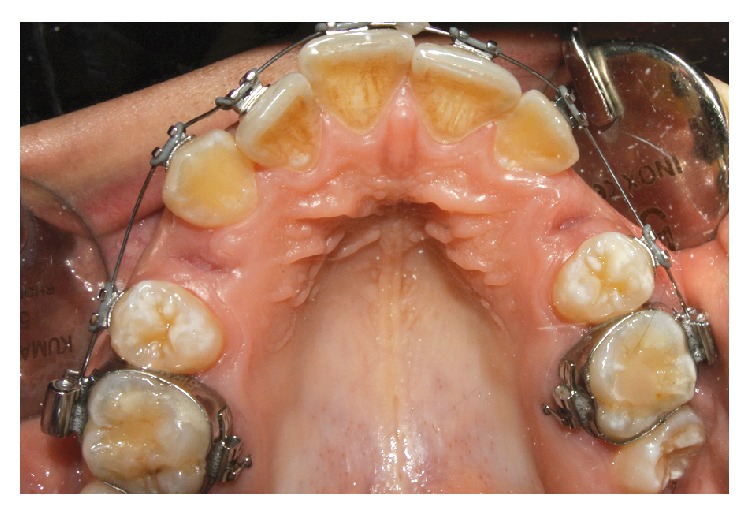
At 21 days after surgery, both postextraction healing sites were completely closed with the soft tissues.

This study was designed to test the efficacy of L-PRF in fostering socket healing after tooth extractions. The 26 split-mouth case control extractions that constituted our study were statistically enough to prove the ability of L-PRF to improve the early healing phases (hemostasis and epithelial closure), reducing the inflammatory process and the risk of infection. The reported results of the experimental sites showed, in the first 7 days after the tooth extractions, a fast evolution of the healing and a positive effect on pain. After a week, minor differences between the two groups are reported (Figures 6 and 7). These effects could be related to the biochemical and structural features of the L-PRF [29], which collects a large quantity of leukocytes (about 60% of the initial blood harvest) and platelets embedded in a fibrin matrix [30, 31]. The fibrin architecture of L-PRF, constituted by connected trimolecular junctions (or equatorial), due to a slow polymerization of the platelet concentrate and due to the absence of heterologous thrombin, induces a flexible fibrin network, able to promote the gradual release of growth factors and leukocytes migration. The fibrin membrane promotes the mechanical protection of the surgical site and, biologically, it interacts with the physiological mechanisms of healing favoring the angiogenesis [13, 14]. The fibrin induces the expression of *α*v-*β*3 integrin by endothelial cells, allowing the links with structural proteins, such as fibronectin and vitronectin, supporting the process of formation of capillaries [12]. In relation to the previous properties, the fibrin also allows the association of some growth factors, such as FGFb (fibroblast growth factor basic) and PDGF (platelet-derived growth factor) involved in the angiogenic process and useful as chemotactic factors, favoring diapedesis of white blood cells [12]. The immunological properties of the L-PRF, resulting from its content in leukocytes, could be useful to prevent the surgical site infections, such as postextraction alveolitis, with a consequent reduction of the inflammation symptoms. The presence of leukocytes is a very important parameter to stimulate healing and wound control [32].

The main limitation of this exploratory study was that the extraction sites were voluntarily very simple, with all alveolar walls preserved. It allowed standardizing the study easily to reach a very clean result, but it does not reflect the real strength and advantages of L-PRF. This material is particularly useful and efficient in complex situations, when some walls are destroyed and the bone regeneration is difficult, but an accurate split-mouth study with this kind of cases is virtually impossible to standardize. It is however the needed next step of evaluation and validation of the use of L-PRF during tooth extractions.

## 5. Conclusion

Even if the selected samples are limited, the reported results suggested that the use of L-PRF in postextraction sockets filling is an efficient and useful procedure in order to manage the postoperative pain and to enhance the alveolar soft tissue healing process, especially in the first days after the extractions, reducing the early adverse effects of the inflammation. This study represents a preliminary clinical trial, which could be used as baseline for further histologic studies.

## Figures and Tables

**Table 1:** 

Number of patients	Number of paired extractions per patient	Tooth extracted
10	1	Premolar
7	2	Canine/premolar/molar
6	3	Canine/premolar/molar
3	4	Canine/premolar/molar

**Table 2:** 

Healing Index	3 days	7 days	14 days	21 days
L-PRF	4.8 ± 0.6	4.5 ± 0.5	4.2 ± 0.2	4.1 ± 0.1
CTRL	5.1 ± 0.9	4.9 ± 0.3	4.3 ± 0.3	4.2 ± 0.2
*P*	0.197	0.05	0.01	0.0002

L-PRF = study site; CTRL = control site.
